# A New Efficient Approach to Simulate Material Damping in Metals by Modeling Thermoelastic Coupling

**DOI:** 10.3390/ma15051706

**Published:** 2022-02-24

**Authors:** Christin Zacharias, Carsten Könke, Christian Guist

**Affiliations:** Institute of Structural Mechanics, Bauhaus-University Weimar, 99423 Weimar, Germany; carsten.koenke@uni-weimar.de (C.K.); christian.guist@uni-weimar.de (C.G.)

**Keywords:** thermoelastic damping, finite element simulation, modal damping, dynamic simulation, experimental damping measurement

## Abstract

The realistic prediction of material damping is crucial in the design and dynamic simulation of many components in mechanical engineering. Material damping in metals occurs mainly due to the thermoelastic effect. This paper presents a new approach for implementing thermoelastic damping into finite element simulations, which provides an alternative to computationally intensive, fully coupled thermoelastic simulations. A significantly better agreement between simulation results and experimental data was achieved, when compared with the empirical damping values found in the literature. The method is based on the calculation of the generated heat within a vibration cycle. The temperature distribution is determined by the mechanical eigenmodes and the energy converted into heat, and thus dissipated, is calculated. This algorithm leads to modal damping coefficients that can then be used in subsequent analyses of dynamically excited oscillations. The results were validated with experimental data obtained from vibration tests. In order to measure material damping only, a test setup excluding friction and environmental influences was developed. Furthermore, comparisons with fully coupled thermoelastic simulations were performed. It was clear that the new approach achieved results comparable to those of a computationally expensive, coupled simulation with regard to the loss factors and frequency response analyses.

## 1. Introduction

The simulation of dynamic processes is essential in almost every engineering discipline, including structural and mechanical engineering, aerospace engineering and micro- and nanotechnology. Besides the stiffness and mass of a structure, the accurate modeling of damping behavior is highly important to predict amplitudes and frequencies correctly, to avoid resonance phenomena, etc. Nevertheless, the estimation of damping parameters is challenging. Several mathematical models have been established to consider energy dissipation, e.g., viscous damping or Rayleigh damping. Although these assumptions are sufficient in many applications, they can lead to inaccurate predictions. Therefore, in practice, it is often necessary to perform experimental vibration analyses in addition to the computational development process. To avoid this effort, there is a need for precise and fast usable damping models.

One reason behind the difficulty in modeling energy dissipation realistically lies in the diversity of sources. In the considered structures, three main causes can be distinguished. Within the material, energy is dissipated through physical processes (material damping). At the levels of bearings, suspensions and connecting joints, dissipating friction occurs (joint damping). Furthermore, mechanical vibrations are attenuated by the interaction with the environment (air or radiation damping).

This paper focuses on material damping. In recent years, material damping has become increasingly important in developing and improving advanced materials and structures. High intrinsic energy dissipation guarantees favorable vibration and acoustic behavior. Examples are carbon-fiber-reinforced composites, e.g., those presented by Xu et al. [1] or Attard et al. [2], and sandwich panels, as described by Ledi [3]. Special honeycomb sandwich panels are outlined, e.g., by Arunkumar et al. [4] and Sadiq et al. [5]. There are several factors influencing the material damping discussed in the literature, for example the orientation of the fibers in fiber-reinforced composites (see [6,7,8]) or the manufacturing. Wesolowski et al. studied the material damping in laminated composite structures in comparison with conventional materials [9]. We present a simplified and convenient method for the simulation of material damping of homogeneous materials in the framework of finite element modeling. The main physical effect causing energy dissipation within the material is thermoelastic relaxation. Depending on the microstructure and the environment, other sources of damping can be dislocations, friction on grain boundaries, electromagnetic effects, etc.

In a thermoelastic solid there is a coupling effect between the elastic strain field and the temperature field. Therefore, mechanical vibrations cause temperature variations. In compressed areas, the temperature increases and, for areas in tension, it decreases. The resulting gradient affects irreversible heat fluxes followed by an increase in entropy and a transformation of mechanical energy into heat.

The temperature gradient equalizes within a characteristic relaxation time, $\tau_{i}$. It is the reciprocal of the thermal peak frequency, i.e., the eigensolution of the heat conduction equation.

(1) $$\tau_{i}=\frac{1}{\omega_{i}}$$

**Figure 1:**
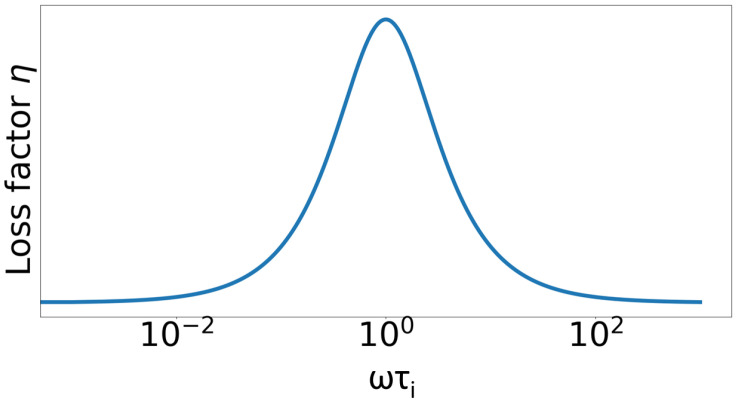
Dependency of the loss factor on the excitation cycle frequency ω.

The amount of thermoelastic damping corresponds to the mechanical eigenfrequency of the structure. The highest loss factor is achieved if the relaxation time approximately coincides with the reciprocal of the natural frequency (see Figure 1). In this case, the period length is exactly sufficient to bring the system back to equilibrium by heat flows.

Research on thermoelastic coupling has a long history and goes back to the work of Duhamel in 1837 [10], as described in [11]. In 1956, Biot provided a mathematical formulation of thermoelastic materials and solution techniques [12]. These theories were extended and refined in the following years and established in standard books, e.g., by Parkus [13] or Nowacki [14]. Over several years, intensive research has emphasized the development of simplified analytical calculations of thermoelastic damping.

In 1937, Zener [15,16,17] published a series of seminal articles concerning energy dissipation in thin bending beams with one-dimensional heat conduction. The author developed a well-established formula to calculate the loss factor η.

(2) $$\eta =\frac{\alpha^{2}ET_{0}}{\rho C_{p}}\frac{\omega \omega_{i}}{\omega^{2}\omega_{i}^{2}}$$

The first fraction describes the material-dependent damping potential, the so-called relaxation strength, where α= the thermal expansion coefficient, E= Young’s modulus, $T_{0}=$ the temperature of the solid, $C_{p}=$ the heat capacity under constant pressure and ρ= density. The second fraction of the formula is frequency-dependent with the current circular frequency, ω. Zener developed the expression for thermoelastic damping containing an infinite series of the thermal frequencies, $\omega_{i}$, and the corresponding thermal modes. He showed that, in thin beams, only the first thermal frequency is relevant and simplified the model to the above-mentioned formula. The thermal peak frequency $\omega_{i}$ is calculated using $C_{p}$, the thermal conductivity, λ, the density, ρ, and the thickness of the beam, *h*.

(3) $$\frac{1}{\omega_{i}}=\tau_{i}={\left(\frac{h}{\pi }\right)}^{2}\frac{C_{p}\rho }{\lambda }$$

Zener showed that the restriction to the first thermal mode is sufficient in the case of thin bending beams.

Some years later, Chadwick [18,19] and Alblas [20,21] extended Zener’s approach and applied it to general three-dimensional solids. In a more recent publication, Lifshitz et al. [22] revised the thermoelastic beam theory and refined the solution by also using higher thermal eigensolutions. Their formula has been used as basis in several further studies (e.g., [23,24,25]).

In general, two approaches for calculating thermoelastic damping can be distinguished, namely, dissipated mechanical energy as the imaginary solution of thermoelastically coupled elastic equation or as generated entropy due to temperature gradients (see Section 2.1). In this paper, we focus on the entropy method. Concerning this topic, Kinra et al. [26] and Bishop et al. [27,28] derived a calculation based on the second law of thermodynamics. Duwel et al. [29] formulated a strongly (two-way) coupled and a weakly (one-way) coupled approach to calculate thermoelastic dissipation in microresonators and presented experimental results. Chandorkar et al. [30] emphasized the superposition of mechanical and thermal modes to consider thermoelastic relaxation. Hao et al. [31] extended the approach to anisotropic materials and embedded the calculation in a finite element algorithm. Tai et al. [32,33] developed simplified concepts for beams and plates based on the entropy theory.

Most of the cited literature refers to micro- and nanoresonators, since thermoelastic damping is often the dominant loss mechanism in microstructures [34]. In macrostructures, thermoelastic damping can be a significant loss mechanism, especially in thin-walled structures. For example, Cagnoli [35] showed this in experiments and calculations on thin circular disks. Concerning nonlinear damping models, the recent publications by Huang et al. [36], as well as Amabili [37], should be mentioned.

Even if other damping mechanisms, especially joint damping, take a decisive role in complex structures, the calculation of material damping is an important component for the realistic prediction of the dynamical behavior.

The described analytical approaches allow exact calculations of thermoelastic damping to be conducted, but are not suitable for complex geometries. Serra et al. [38] developed a finite element formulation for thermoelastically coupled problems. The authors applied the underlying theory on shell and solid elements that are characterized by coupled damping and stiffness matrices and considered thermoelastic damping. These elements showed a good agreement with the experimental data in statistical analyses and dynamic simulations requiring direct time-integration methods. The significant disadvantage, in this case, is the enormous computational effort for fully coupled thermoelastic simulations.

We present an approach that allows the calculation of thermoelastic damping to be performed based on the stress and strain distribution in the mode shapes. Using an entropy method, modal damping coefficients were determined and applied in finite element simulations of dynamically excited components in the time and frequency domain. This allowed us to consider material damping in time-efficient simulations. Furthermore, the damping coefficients of specific mode shapes were measured in physical experiments to verify the simulated results.

## 2. Materials and Methods

### 2.1. Simulation

**Figure 2:**
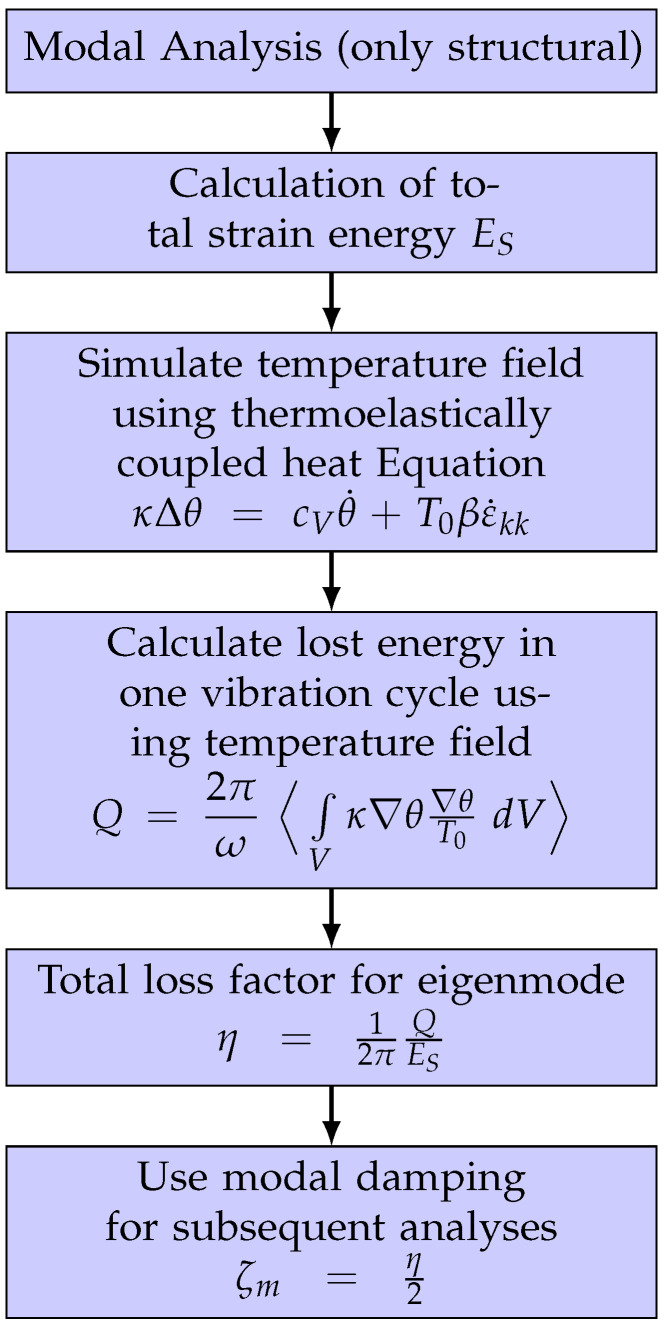
Flow chart for numerical solution procedure.

Since Zener’s first publications in the 1930s [15,17], there have been two main approaches to interpreting thermoelastic damping, explained as follows:

- Dissipated mechanical energy: The calculation of thermoelastic damping is based on the phase lag between stresses and corresponding strains. The thermoelastically coupled differential equations of elasticity and heat conduction have to be solved in the complex domain. The loss factor is equal to the ratio of the imaginary and real parts of the solution.
- Entropy approach: The amount of dissipated energy is equal to the heat generated during the elastic vibration. The energy transferred into heat can be calculated by analyzing the heat flows in the structure causing an increase in entropy. The loss factor is obtained from the quotient of dissipated energy to total strain energy. This approach is used in the present paper. The general procedure of the calculation is shown in the flowchart in Figure 2.

The amount of generated heat during one vibration cycle is determined starting from the rate of entropy of a solid with volume, *V*.

(4) $$\dot{S}=\int_{V}\frac{-\nabla q}{T}\;dV=-\int_{V}\nabla \left(\frac{q}{T}\right)\;dV+\int_{V}q\nabla \frac{1}{T}\;dV$$

where *S* denotes the entropy, q is the vector of heat flux and *T* is the absolute temperature of the solid. Following the derivation in [39], the equation can be transformed using the divergence theorem. If the terms are converted into a surface integral, the first integral becomes zero. The vibration is defined to be adiabatic, i.e., there is no heat flux over the boundaries of the solid.

(5) $$\dot{S}=\int_{V}q\nabla \frac{1}{T}\;dV$$

The absolute temperature, *T*, is defined as the sum of the reference temperature, $T_{0}$, and the temperature increment, θ. The expression 1/T is replaced by its Taylor expansion in the vicinity of the reference temperature. Under the assumption of very small temperature variations in the present thermoelastic problems, the first term produces a zero integral.

(6) $$\dot{S}=\int_{V}q\nabla \left(\frac{1}{T_{0}}-\frac{\theta }{T_{0}^{2}}\right)\;dV\approx \int_{V}-q\nabla \frac{\theta }{T_{0}}\;dV$$

The same assumption allows us to calculate the rate of produced heat using the reference temperature. Using Fourier’s law

(7) q=−κ∇θ

to substitute q, the following formula is obtained:

(8) $$\dot{Q}=\int_{V}\kappa \nabla \theta \frac{\nabla \theta }{T_{0}}\;dV$$

with the thermal conductivity κ. Considering one vibration cycle from t=0 to t=T,

(9) $$Q=\frac{2\pi }{\omega }\;\langle \int_{V}\kappa \nabla \theta \frac{\nabla \theta }{T_{0}}\;dV\rangle$$

where $\langle f\left(x\right)\rangle =\frac{1}{T}\int_{0}^{T}f\left(t\right)\;dt$ denotes the time average of the function. The thermoelastic loss factor, η, is defined as the ratio of generated thermal energy, *Q*, to total strain energy, $E_{S}$.

(10) $$\eta =\frac{1}{2\pi }\frac{Q}{E_{S}}$$

The loss factor of a component modeled with three-dimensional solid elements is obtained in a procedure combining several numerical solution steps. A loss factor is calculated for each eigenmode in order to apply modal damping in subsequent analyses. For this purpose, a purely structural natural frequency analysis is run first. From the eigensolutions, the (normalized) strain distributions are calculated.

The coupling of elastic strain field and temperature field is mathematically described by the thermoelastically coupled heat equation, e.g., as derived by Biot in 1956 [12].

(11) $$\kappa \Delta \theta =c_{V}\dot{\theta }+T_{0}\beta {\dot{\varepsilon }}_{kk}$$

where $\beta =3\frac{\alpha K}{c_{V}}$ is defined as the thermoelastic coupling constant with the elastic bulk modulus *K* and the thermal expansion coefficient α. It describes the relation between an adiabatic volume change and the temperature variation in a solid.

In the coupled heat equation, the mechanical volumetric strains, $\varepsilon_{kk}$, act as a source term. Equation (11) is solved numerically in the time domain to obtain the temperature variations in the solid over one vibration cycle.

For the solution, the software package FEniCS was used. Therefore, the problem was implemented in a finite element environment, based on the weak form of the coupled differential problem. The time derivatives were discretized by a finite difference algorithm [40].

(12) $$\begin{array}{cc} \dot{\theta } & =\frac{\theta^{n+1}-\theta^{n}}{\Delta t} \end{array}$$

(13) $$\begin{array}{cc} {\dot{\varepsilon }}_{kk} & =\frac{\varepsilon_{kk}^{n+1}-\varepsilon_{kk}^{n}}{\Delta t} \end{array}$$

The temperature is calculated using the thermoelastically coupled heat equation. Once the temperature has been determined, the loss factor can be calculated using Equations (9) and (10). In the approach described, a weak thermoelastic coupling is used, i.e., the elastic strain field influences the temperature distribution, but not vice versa.

The calculated loss factor, η, is specific for an eigenmode. In the mode-based subsequent analyses, they can be used as modal damping values, $\zeta_{m}$.

(14) $$\zeta_{m}=\frac{\eta }{2}$$

### 2.2. Material

**Figure 3:**
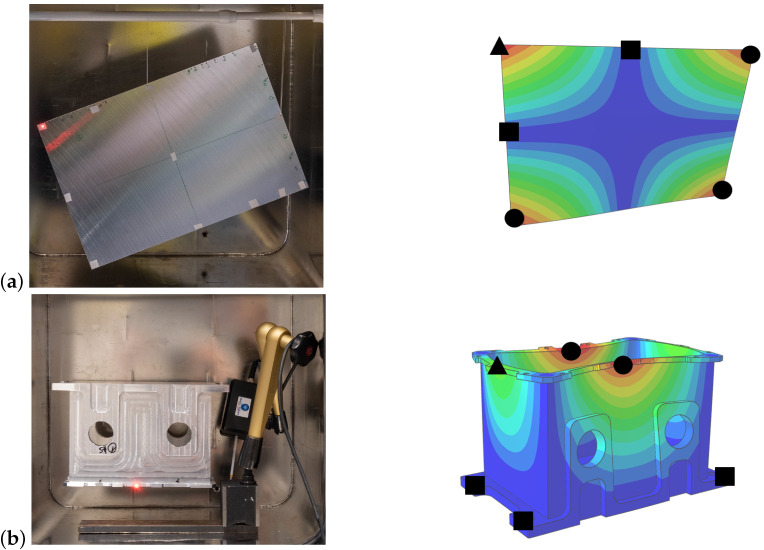
Experimental setup and positions of suspension (■), measurement points (●) and excitation points (▲—measurement and excitation) on the first three eigenmodes of the rectangular aluminum plate (**a**) and the box component (**b**).

In the experimental studies, two types of specimens were considered. First, a simple plate geometry was investigated to validate the general procedure and verify the simulation results. In a second step, the method was extended to a more complex three-dimensional geometry. This was designed in the style of a simplified gearbox case. Both components are shown in Figure 3.

In order to ensure comparability, both bodies were made of the same aluminum alloy (AlMg4.5Mn0.7). The material parameters are listed in Table 1.

### 2.3. Experimental Studies

The experiments aimed to measure the material damping. Therefore, other sources of energy dissipation had to be eliminated. To avoid air damping effects, the experimental setup was located within a vacuum chamber. With the available device, a pressure of approximately 5mbar was achieved. All the processes during the experiments had to be controllable from the outside.

Furthermore, the impact of joint damping, which occurs as friction on bearings, suspensions and joints, had to be eliminated. Friction arises due to relative movements; therefore, the displacement at bearings should be inhibited. In the experiment, the specimens were suspended on very thin elastic strings that were placed in the zero lines of the mode shapes to simulate a free bearing. Therefore, a new experimental setup had to be taken for each eigenmode. Two examples for bearing and excitation points are shown in Figure 3. For a better understanding of the shown eigenmodes, video simulations are included in the Supplementary Material Videos S1 and S2.

The excitation was also designed in such way to avoid additional damping sources. Therefore, the specimens were excited with an automatic impulse hammer, which guaranteed a minimum of contact. The hammer was installed within the vacuum chamber and controlled from the outside.

As a result, the velocity of a single point was measured. The recording of the data was also performed in a contactless manner using a laser Doppler vibrometer.

The velocity over time was measured and analyzed with an algorithm based on LabView software. By applying an exponential curve fit on the decay function, the damping coefficient, ζ, was determined. This measure could be transformed easily into other damping characteristics, e.g., the loss factor, η.

## 3. Results

### 3.1. Rectangular Plate

**Figure 4:**
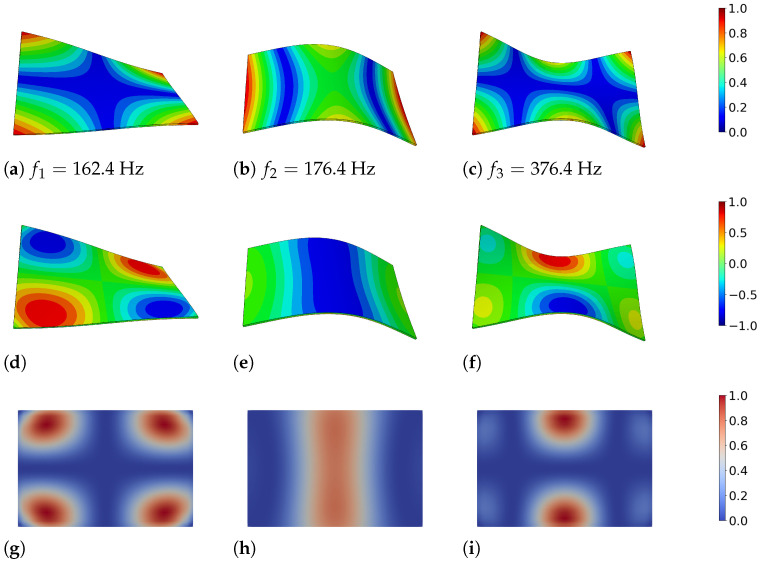
(**a**–**c**) Mode shapes of the rectangular aluminum plate 300 mm × 200 mm × 3 mm, deflection normalized to 1. (**d**–**f**) Distribution of hydrostatic pressure at the surface of the plate, $\sigma_{kk}=\sigma_{xx}+\sigma_{yy}+\sigma_{zz}$. (**g**–**i**) Spatial distribution of the dissipated energy, *Q*, in the first three eigenmodes of the plate at time T/4. A cut at mid-plane level of the plate is displayed. The values were normalized to 1 with respect to the maximum in each mode since they were based on relative displacements and temperature fields.

Figure 4 provides the correlation among the magnitude of displacement of the plate structure *u*, the hydrostatic stress distribution $\sigma_{kk}$ and the generated heat *Q* in a specific mode shape. Since the eigenmodes are normalized vectors, the amplitudes were not significant and all quantities were scaled to 1 in the figure. The vibration of the plate follows a sine curve over time and the results are shown at time t=T/4, i.e., at the time step of maximum deflection, maximum stress level and maximum thermoelastic heat generation. Furthermore, Figure 4g–i display a cut on the mid-plane level of the plate because the heat flowed in the thickness direction. Therefore, the highest amount of energy was dissipated in the mid-level. The heat flow at the surface was negligible.

From the figure, it is apparent that the generated heat (and therefore the dissipated energy) correlated directly with the spatial distribution of the hydrostatic stress. The thermoelastic coupling only affected those parts of the stress or strain tensor that were associated with a volume change. Locations with a high hydrostatic stress level show large heat production.

**Figure 5:**
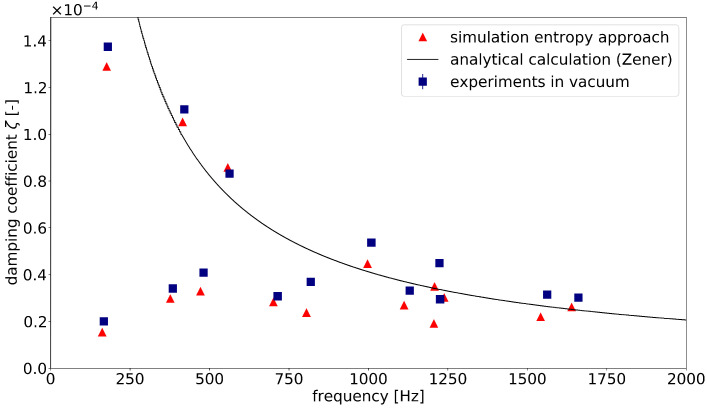
Comparison of experimental data, simulated damping coefficients and analytically calculated values with equation according to Zener [15].

Figure 5 presents the experimental data and the simulation of the loss factors. For comparison, the analytical calculation according to Zener for a specimen thickness of 3 mm is shown. The experiments were conducted on four individual plates that were identical in construction and four measurement points on each plate were used. The results present an average of the results per eigenfrequency. The standard deviation is shown as bars; however, in the case of the simple plate, the value was too small to be visible.

Comparing the two data series, it can be seen that the loss factors measured experimentally were slightly higher than the simulated data. This discrepancy may be explained by the experimental challenges. Every disruptive factor in the experimental setup caused an increase in damping. Due to the very low magnitude of the loss factors measured in this study, the experiments were very sensitive to inaccuracies. The available vacuum chamber reached only a rough vacuum of ≈ 5 mbar. The remaining air resistance led to a small increase in the damping coefficient. Furthermore, the suspension was due to practical reasons realized at the edge of the plate. Therefore, it did not always matched exactly the zero line of the mode shape. This also affected the measured damping due to joint friction. Overall, the results of the simulations match those of the experiments well. Especially, the relative differences between the mode shapes could be represented very well.

As mentioned above, Serra et al. [38] developed a fully coupled finite element formulation considering thermoelastic loss. This theory was implemented in the FE-Software package ANSYS as SOLID226, SOLID227 or PLANE223 elements. These element types are available for full transient (time–domain) and full harmonic (frequency–domain) analyses.

Both components studied in the present contribution were simulated with these elements to compare the fully coupled approach with the simplified method presented here. The plate was discretized with SOLID226 elements (20-node hexahedrons) with an element size of 2 mm and 4 layers of elements in the thickness direction. A full harmonic analysis was performed with a point load applied sinusoidally at the corner point of the plate in the out-of-plane direction. No further boundary conditions were defined to simulate a free suspension.

The results of each frequency step include a real and an imaginary part of the solution. In order to calculate the loss factor, the complex data for the total strain energy were extracted per element and summed up over the whole structure. The loss factor is defined as the quotient of imaginary and real strain energy.

(15) η=Im(ES)Re(ES)

**Figure 6:**
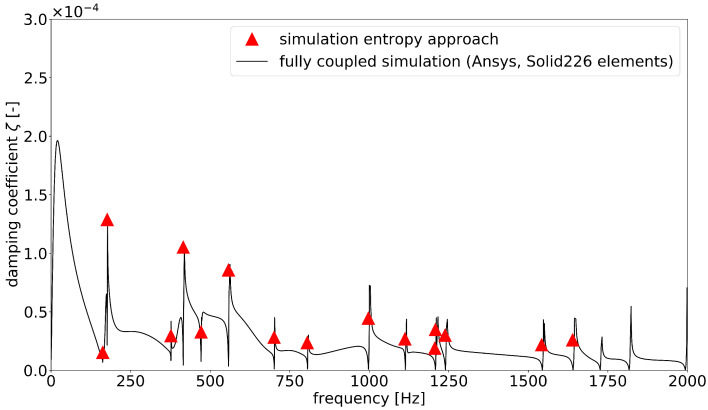
Comparison of the damping coefficients of the modal-based entropy approach with the fully coupled thermoelastic ANSYS simulation for the rectangular plate.

Figure 6 provides the results obtained from the ANSYS analysis in comparison to the loss factors calculated with the simplified approach. It is apparent from the diagram that the simplified energy approach produced only sample points at the resonant frequencies, whereas the outcome of the fully coupled harmonic response analysis was one loss factor per frequency step. If the excitation frequency in the simulation matches exactly the eigenfrequency of the plate, there is a pole in the curve. Therefore, the absolute height of the peaks depends on the frequency discretization and is not representative. The calculated loss factors followed the course of the graph satisfactorily. Both the magnitude and the differences in the mode-dependent damping factors could be represented accurately.

### 3.2. Complex Three-Dimensional Geometry

**Figure 7:**
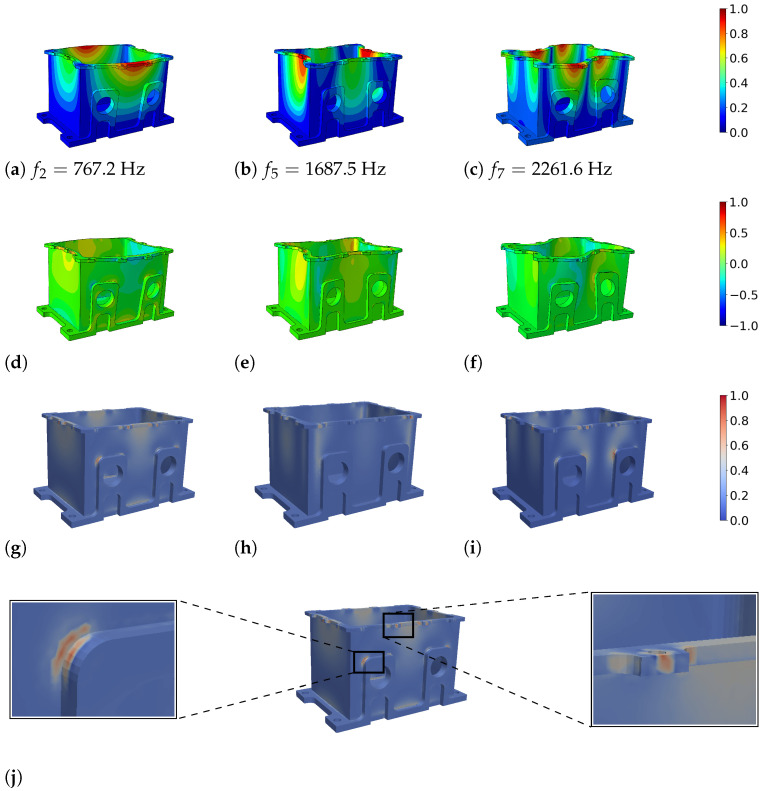
(**a**–**c**) Mode shapes 2, 5 and 7 of the box component; displacement magnitude normalized to 1. (**d**–**f**) Distribution of hydrostatic pressure $\sigma_{kk}=\sigma_{xx}+\sigma_{yy}+\sigma_{zz}$. (**g**–**i**) Spatial distribution of the dissipated energy, *Q*, at time T/4. The values were normalized to 1 with respect to the maximum in each mode since they were based on relative displacements and temperature fields. (**j**) Detailed view of locations with high energy dissipation.

Figure 7 shows the correlation of the magnitude of displacement, *u* (a–c), the hydrostatic stress, $\sigma_{kk}$ (d–f), and the generated heat, *Q* (g–i), in a specific mode shape for the complex geometry. The eigenmodes 2, 5 and 7 were chosen as examples. Just as in Figure 4, all data were scaled to 1. The heat conduction, *Q* (or, equivalently, the dissipated energy), was plotted at t=T/4. The points of maximal heat generation did not occur on the faces of the box but at the corners and edges, where the stress peaks were located. This is shown graphically in the detail plot under (j). If high stresses and strains occurred locally due to geometry reasons, there was a large difference in stress in respect to neighboring areas. This was followed by a temperature gradient with short paths of heat conduction, which led to locally high energy dissipation.

**Figure 8:**
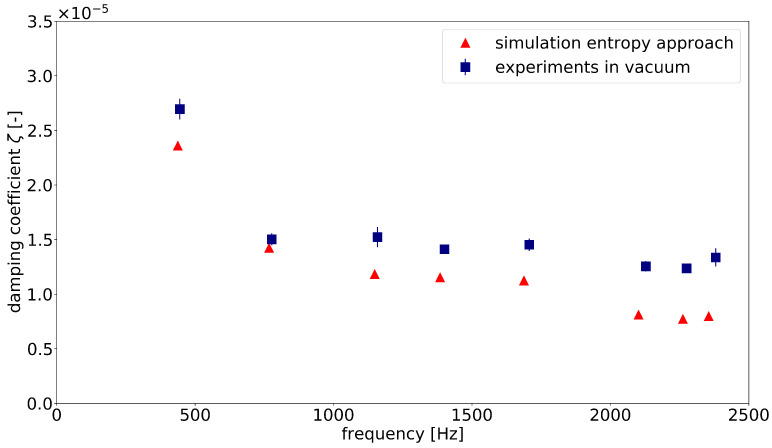
Comparison of the experimental data and the damping coefficient determined by the entropy approach for the box component.

Figure 8 compares the experimental values of the damping coefficients ζ with the simulated data obtained using the entropy method. The experiments were performed on two identical components and two measurement points were used for each eigenmode. The values shown are averaged measurement results. The standard deviation is represented by bars. The damping coefficient of the first eigenmode had a higher value than the following one. The other coefficients remained on the same level, with a slightly decreasing trend. This course was consistent with both calculated and measured values. Normally, the experimentally measured values are always slightly higher than the calculated ones (except in the second eigenmode). This is mainly due to the systematic problems in the experimental implementation, as already explained in Section 3.1. For practical reasons, all samples were suspended in the holes at the bottom of the case. These locations were not deformed in all the eigenmodes considered. However, they were not always the exact zero lines of the system, so there may have been additional effects due to frictional damping. In addition, high vacuum could not be achieved with the available vacuum chamber. During the experiments, air pressure of about 5 mbar was always maintained, which led to a slight air resistance during the oscillation. Taking these facts into account, there was overall good agreement between simulation and experiment.

**Figure 9:**
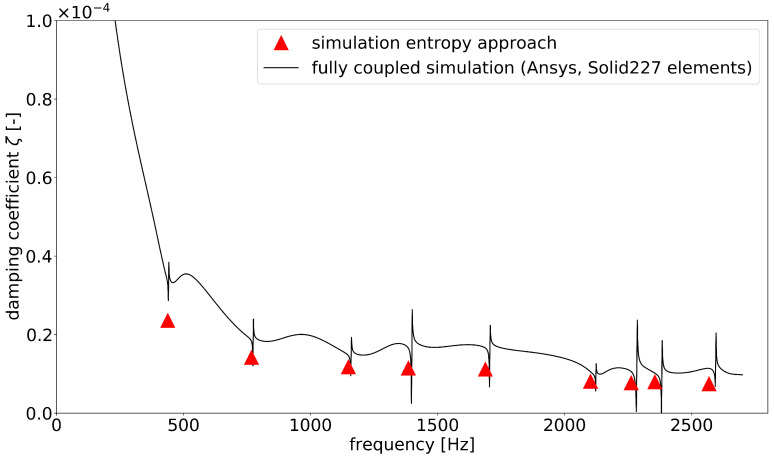
Comparison of the damping coefficients of the modal based entropy approach with the fully coupled thermoelastic ANSYS simulation for the box component.

Figure 9 exhibits the damping coefficients calculated by the entropy approach with the damping coefficient curve extruded from an ANSYS harmonic simulation with discretization by SOLID227 elements (fully coupled thermoelastic tetrahedron elements). In the full harmonic simulation, the system response was calculated for each frequency step. Afterwards, the loss factor was obtained by dividing the imaginary part of the strain energy by the real part of the strain energy. Therefore, one data point was calculated for each frequency step. The results of the entropy simulation were determined as discrete damping coefficients at the resonant frequencies. The curve of the fully coupled simulation had poles at the resonant frequencies; therefore, an exact comparison is difficult. The damping values calculated by the entropy approach followed the course of the curve satisfactorily.

**Figure 10:**
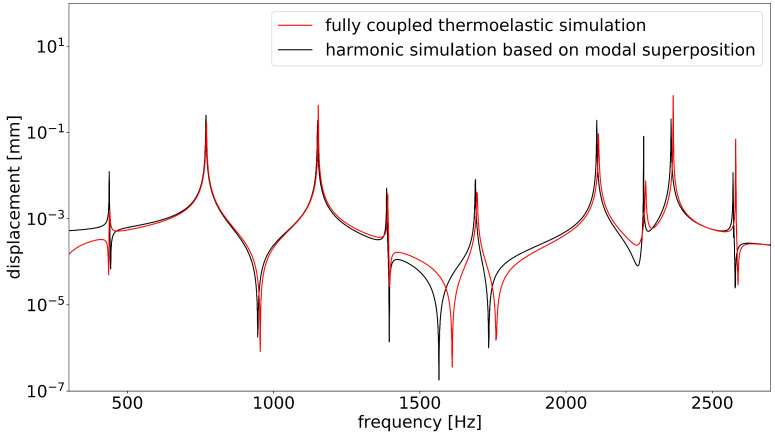
Comparison of displacement in y-direction of single node. Red curve: fully coupled simulation with thermoelastic elements SOLID226. Black curve: harmonic simulation based on modal superposition, use of modal damping coefficients that were calculated with the entropy approach.

**Figure 11:**
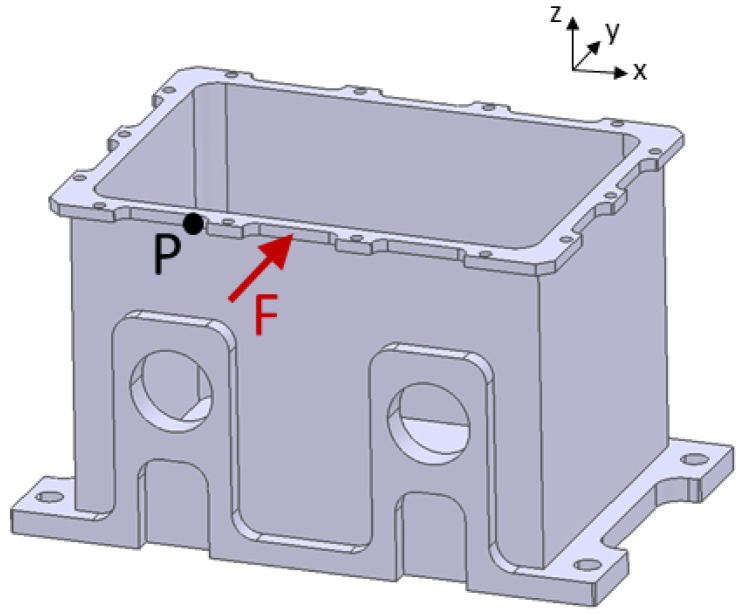
Excitation point of force F and measurement point P of displacement in frequency–domain analysis.

In the next step, the calculated damping coefficients were applied in a frequency–domain simulation based on modal superposition. For this purpose, the discretely determined damping coefficients were used as modal damping ratios. Figure 10 shows a comparison of a single-point displacement in the modal-based harmonic simulation with the same displacement component in the full harmonic simulation. For this analysis, a single load F in the y-direction was applied harmonically at a point in the middle of the upper edge of the component, as shown in Figure 11. The displacement in the y-direction was determined at a node denoted by P. The course of the curves and the width of the peaks agreed satisfactorily. The absolute height of the peaks was not significant because it depended on the frequency discretization. Only at eigenfrequency 5 at approximately 1687 Hz were there inaccuracies. In this eigenmode, the considered node was close to a zero line (see Figure 7b), so that the oscillation mode could not be reproduced well in both simulations. This led to distortions of the results.

Note that only frequencies f > 200 Hz are displayed here. The first natural frequency occurred at 437 Hz, i.e., the relevant frequency domain was covered. No boundary conditions were applied to the model to ensure comparability with the experiment. Therefore, a large influence of the rigid body modes occurred at low frequencies in the full harmonic simulation.

Overall, the excellent agreement of the curves shows that, with the approach presented here, results equivalent to those of a fully coupled calculation could be achieved with a significant reduction in computing time.

## 4. Discussion

**Figure 12:**
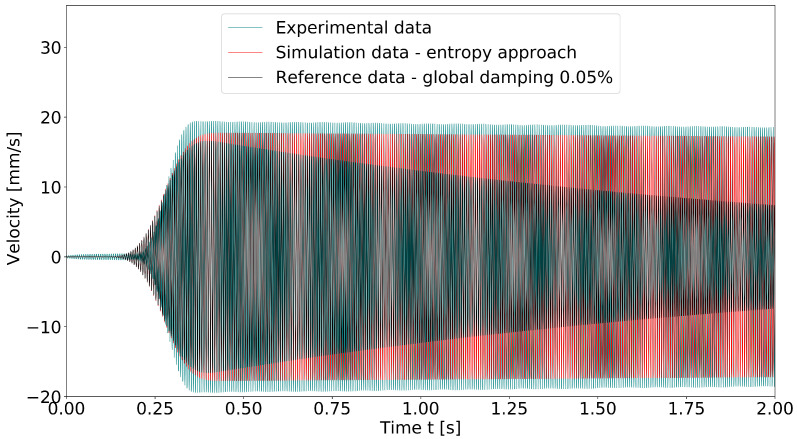
Comparison of velocity time series of impulse-excited plate structure: experiment, simulation with global structural damping and simulation with modal damping. The velocity was measured/analyzed on a corner point of the plate.

**Figure 13:**
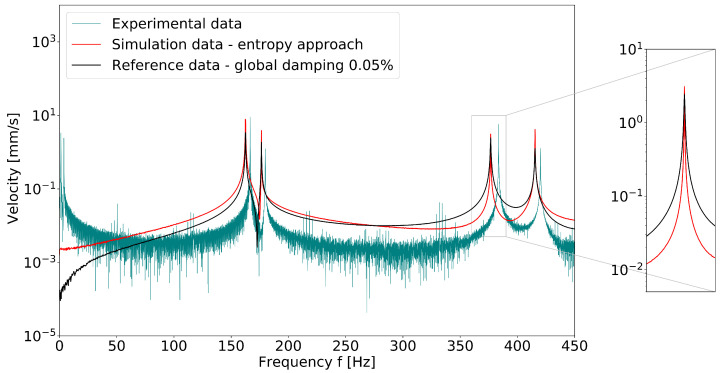
Comparison of velocity frequency spectrum of impulse-excited plate structure: experiment, simulation with global structural damping and simulation with modal damping. The velocity was measured/analyzed on a corner point of the plate.

First, the results demonstrate that thermoelastic damping is clearly mode-dependent. The damping coefficient of the aluminum alloy considered was in the range from $1\times 10^{-5}$ to $1\times 10^{-4}$, but the values of the individual mode shapes of a component differed from each other by a factor of 4 or more. In the literature, one general loss factor or damping coefficient is often given for a material (see, for example, [41,42]). Usually, it is not sufficient to use a global damping value for the simulation, as illustrated by Figure 12 and Figure 13. In these diagrams, the system response of the rectangular aluminum plate in the first eigenmode to an impulse excitation is shown. Both time and frequency domain data were taken at a corner point of the plate. Figure 12 shows the time series of the velocity, filtered to the first natural frequency (band pass filter 160 Hz–170 Hz). The experimental data were compared with the finite element simulation using a global damping factor of 0.05% (following [41], where 0.04% was proposed). Furthermore, the results were set against the finite element simulation using the modal damping determined by the entropy approach presented here. It was obvious that the assumption of a global damping coefficient led to a decay curve that deviated strongly from the experimentally measured time course. Figure 13 shows the same system response in the frequency domain. The amount of damping is represented by the width of the peaks. The assumed global damping of 0.05% agreed well with the calculated damping coefficient of the second eigenmode, which is why the curves overlapped well in this range. A possible explanation for this might be that the second eigenmode was close to a pure beam bending; therefore, it could be calculated with Zener’s theory. In the other eigenmodes, especially at the third natural frequency at approximately 376 Hz, the spectrum showed the deviation in the width of the peaks.

In comparison with the fully coupled simulations, good accuracy was achieved in the calculation of the loss factors, as could be shown with both components. The comparisons shown in Figure 6, Figure 9 and Figure 10 demonstrate good agreement. At the moment, it is not possible to apply the fully coupled element formulations for finite element analyses based on modal superposition. Therefore, especially for large systems, enormous computation times is required, since the thermoelastically coupled system of equations must be solved in every time or frequency step. The calculation of the system response in the frequency range under harmonic excitation took almost 6 days for the box component (2700 frequency steps). This procedure is usually not suitable for practical use. The frequency response analysis based on modal position using modal damping coefficients offers a great advantage in terms of computing time. Taking all the analytical steps into account, the system response for the box component could be solved in about 2 h. In addition, for the simpler system of the plate, the computation time could be reduced by more than half (5000 frequency steps). The computing times are displayed in Table 2. The bottleneck in the analysis remained the determination of the modal damping coefficients by the method presented here. This calculation step was the only one that was performed on a local computer and was not parallelized, so there is still potential for improvement in this respect. Furthermore, the disadvantages of a mode superposition method in finite element simulation must also be taken into account; the coupling effects of the mode shapes could not be represented and the accuracy of the solution depended on the number of considered modes.

In comparison with the simulated values, the experimental studies deliver always slightly higher damping coefficients. A possible explanation could be that, in the thermoelastic simulation, not all physical effects in the material are captured; therefore, lower values are calculated. However, it is more likely that the deviations are caused by the limitations of the experimental setup. In particular, the box component is difficult to install in the vacuum chamber due to its dimensions and weight. Therefore, for example, in our study, compromises had to be made in the design of the bearings. Since the suspension could not always be realized exactly in the zero line of the eigenmode, slight damping effects due to friction occurred. In addition, a complete vacuum was not achieved in the experimental setup, so that a small influence due to air damping could be recorded. The experimental setup should be improved so that measured values can better compare with numerically simulated ones.

## 5. Conclusions

We here present a method to calculate modal thermoelastic damping coefficients for arbitrary components discretized with solid elements. The analysis is based on an increase in entropy during mechanical vibrations that leads to energy dissipation. Modal damping ratios were calculated and used in subsequent finite element simulations based on modal superposition.

The results were compared with simulations using a thermoelastically fully coupled element approach. It is shown that similar results were obtained for both the loss factors and the system response in the frequency spectrum in significantly lower computation times.

The comparison of the modal damping coefficients with the experimental data showed good agreement. The physically measured damping values are always slightly higher than the calculated ones.

In conclusion, the method seems suitable for calculating material damping. However, in order to make realistic predictions of the damping behavior of structures, other causes of energy dissipation must also be taken into account. Suitable models for friction and air damping must be used in the simulations.

In addition, the extension of the method to other materials might prove an important area for future research. The method was developed and tested on aluminum. As a next step, anisotropic materials or laminated components could be investigated.

Furthermore, interesting research questions can be derived from the possibility to locate the areas of high energy dissipation in the simulation. This provides a good starting point for the optimization of geometries or surfaces.

## Figures and Tables

**Table 1:** Material parameters of the used aluminum alloy.

Parameter	Value	Unit
Young’s modulus *E*	$7\times 10^{10}$	$N/m^{2}$
Poisson’s ratio ν	0.34	
density ρ	2660	$\mathrm{kg}/m^{3}$
thermal conductivity λ	140	W/mK
heat capacity $C_{p}$	904	J/K
thermal expansion coefficient α	$2.31\times 10^{-5}$	$K^{-1}$

**Table 2:** Computing time in CPU hours for a frequency–domain analysis using a direct solution procedure with fully coupled thermoelastic elements and a mode superposition solution procedure with modal damping. The modal damping coefficients were determined with the entropy approach provided in the paper. For the rectangular plate, 5000 frequency steps were analyzed and, for the box component, 2700 frequency steps were analyzed. All simulations (except for the calculation of the modal damping coefficients) were run on 16 cores of a compute node with 2 Intel Xeon Processor E5-2650 v4 CPUs with a base clock of 2.2 GHz and 128 GB RAM.

	Plate	Box Component
number of elements	9600 el.	291,320 el.
fully coupled simulation SOLID226/227	34.8	2234.6
		(≈6 days)
entropy approach SOLID186/187	16.5	35.1
eigenfrequency analysis	1.2	7.5
modal damping coeff.	9.3	17.9
harmonic analysis	6.0	9.7

## Data Availability

Not applicable.

## Supplementary Materials

The following supporting information can be downloaded at https://www.mdpi.com/article/10.3390/ma15051706/s1, Video S1: Simulation of mode 1 of the rectangular aluminum plate. The points of suspension, excitation and measurement are shown. Video S2: Simulation of mode 2 of the aluminum gearbox case. The points of suspension, excitation and measurement are shown.
